# Kinetic and Reaction Pathway Analysis in the Application of Botulinum Toxin A for Wound Healing

**DOI:** 10.1155/2012/159726

**Published:** 2011-11-24

**Authors:** Frank J. Lebeda, Zygmunt F. Dembek, Michael Adler

**Affiliations:** ^1^Integrated Toxicology Division, US Army Medical Research Institute of Infectious Diseases (USAMRIID), 1425 Porter Street, Fort Detrick, MD 21702, USA; ^2^USAMRMC, Combat Casualty Care Research Program, ATTN: MCMR-RTC, 504 Scott Street, Fort Detrick, MD 21702-5011, USA; ^3^Division of Medicine, USAMRIID, 1425 Porter Street, Fort Detrick, MD 21702-5011, USA; ^4^Neurobehavioral Toxicology Branch, Analytical Toxicology Division, US Army Medical Research Institute of Chemical Defense, Aberdeen Proving Ground, Edgewood Area, MD 21010, USA

## Abstract

A relatively new approach in the treatment of specific wounds in animal models and in patients with type A botulinum toxin is the focus of this paper. The indications or conditions include traumatic wounds (experimental and clinical), surgical (incision) wounds, and wounds such as fissures and ulcers that are signs/symptoms of disease or other processes. An objective was to conduct systematic literature searches and take note of the reactions involved in the healing process and identify corresponding pharmacokinetic data. From several case reports, we developed a qualitative model of how botulinum toxin disrupts the vicious cycle of muscle spasm, pain, inflammation, decreased blood flow, and ischemia. We transformed this model into a minimal kinetic scheme for healing chronic wounds. The model helped us to estimate the rate of decline of this toxin's therapeutic effect by calculating the rate of recurrence of clinical symptoms after a wound-healing treatment with this neurotoxin.

## 1. Introduction

As characterized by Hanchanale and coworkers [1], the perception of botulinum toxin A has been transformed “from poison to a healing agent.” Much of our present knowledge about this toxin comes from the toxicological literature. Information concerning botulinum neurotoxin serotypes, molecular structures, substrate specificities, mechanisms of zinc-dependent peptide hydrolysis, ion channel formation, and other detailed topics has been extensively reviewed [2–4]. Of the seven immunologically distinct serotypes (A–F) from *Clostridium botulinum* and several other species, type A is the best characterized and is among the most potent of all toxins. The neurotoxin is initially expressed as a single polypeptide of nearly 1300 amino acid residues (MW ~150 kDa). Crude toxin extracts (MW ranges from ~300 to 900 kDa) contain several nontoxic ancillary proteins that form a complex with the neurotoxin. When ingested, these additional proteins are thought to protect the neurotoxin against austere environments such as those found in the certain regions of the gastrointestinal tract [5]. The neurotoxin is posttranslationally modified to form two chains that are covalently bridged with a disulfide bond. The light (L) chain (MW ~50 kDa) has zinc-dependent proteolytic activity, while the heavy (H) chain contains the translocation and binding domains. Subsequent to binding to specific receptors (SV2) [6] at peripheral cholinergic nerve terminals, the receptor-toxin complex is internalized into a membrane-bound compartment that undergoes a drop in pH. This acidification initiates a series of interrelated reactions. Conformational changes occur that allow the insertion of the H-chain into this compartment's membrane. As a result, the disulfide bond that links the L and H chains is reduced, an ion channel is formed, and the presumed proteolytic active moiety, the L-chain, is translocated into the neuroplasm. When the type A toxin substrate, SNAP-25, is selectively cleaved, synaptic vesicle-mediated neurotransmission is blocked that could eventually lead to fatal paralysis.

Since the 1980s, the therapeutic potential of this toxin has been exploited. Extraocular muscles have been injected with the partially purified neurotoxin as an adjunct or alternative to surgical correction in treating strabismus [7, 8]. The chemodenervation effects of this most poisonous of poisons [9] have been used to relax hyperkinetic striated muscle groups to diminish the effects of dystonia and related diseases [10]. Currently, BOTOX has been approved by the U.S. Food and Drug Administration (FDA) for the following indications: strabismus, blepharospasm, cervical dystonia, upper limb spasticity, maxillary hyperhidrosis, chronic migraine [11], and urinary incontinence [12]. These indications along with the temporary enhancement in the appearance (cosmesis) with BOTOX COSMETIC of moderate to severe wrinkles in adults [13, 14] introduces the theme of muscle immobilization in terms of a desired therapeutic outcome. Immobilization has been characterized as a fundamental principle of wound healing [15]. Advantage has also been taken of this toxin's chemoimmobilization property to improve the healing of wounds.

In contrast to the relatively vast amounts of information regarding this toxin's structure and mechanism of action, the newer, off-label uses for botulinum toxin have been less extensively reviewed. To gain further insight regarding the scope of these efforts, we have gathered and examined biomedical research articles by conducting systematic searches of the relevant literature in PubMed, in a manner similar to that of Steele and Madoff [16]. We have examined studies that range from descriptive observations to randomized controlled clinical trials to obtain more information about the components and processes involved in wound healing and the related time courses of action of botulinum toxin A.

The processes observed clinically on the wound healing effects of the type A toxin are at an early stage of our understanding. This proposal is substantiated by evidence-based reviews that critically evaluate this toxin's effects with different indications [17, 18]. We previously noted [19, 20] that only a few clinical studies have focused on kinetic analyses. Constructing even partial models for the clinically observed effects by this toxin remains a challenge. To advance our understanding, we have selected some of those clinical studies that have examined the timing of this toxin's effects.

## 2. Methods

### 2.1. Literature Searches

botXminer, the botulinum reference tool of clostridial neurotoxin citations in Entrez-PubMed/MEDLINE [20], was initially used to search in article titles, abstracts, and MeSH headings for the words “wound” and “heal” or “healing.” A more extensive list of 29 wound-related keywords was then generated: anal, angiogenesis, collagen, cytokine, fibroblast, fibroblastic, fibrosis, fissure, flap, glycosaminoglycan, heal, healing, hemorrhoid, hemorrhoidectomy, hypertrophic, incision, inflammation, inflammatory, keloid, lesion, repair, scar, scarring, sphincterotomy, surgical, tendon, tensile, ulcer, and wound. This controlled vocabulary was used in the batch mode [21] to search botXminer for additional related citations. Another set of filter terms were used to find time-course-related information about this toxin. This set included 26 terms: clearance, day, decay, decline, delay, diffusion, duration, follow up, frequency, hour, hr, interval, kinetic, latency, minute, month, onset, period, persistence, recurrence, repeat, resistance, sec, time, week, and year.

Within the two lists in the batch matrix search, (*a*_1_, *a*_2_,…, *a*_*m*_) and (*b*_1_, *b*_2_,…, *b*_*n*_), the terms undergo the OR operation (⋃) which can be represented in a general form of (*a*_1_ OR *a*_2_ OR, etc.). In addition, the lists are combined with the AND operation $\left(\overset{}{\underset{}{⋂}}\right)$ [21] which results in a batch query that can be represented by

(1) $$\left(a_{1}\overset{}{\underset{}{⋃}}a_{2}\overset{}{\underset{}{⋃}}\cdots a_{m}\right)\overset{}{\underset{}{⋂}}\left(b_{1}\overset{}{\underset{}{⋃}}b_{2}\overset{}{\underset{}{⋃}}\cdots b_{n}\right).$$

Summaries from the two sets of terms were returned by botXminer in the form of tables, histograms, and lists. Lists of citations were subsequently manually examined. Additional keywords and phrases within the more than 70 downloaded text files were automatically searched with file search assistant (v. 3.1, 2009, AKS-Labs, Raleigh, NC, USA).

### 2.2. Analysis of Kinetic Data

Data from the clinical literature that were analyzed were fitted to an exponential function

(2) $$y=y_{0}+\left(a\right)\exp\left(-k_{\text{decay}}t\right),$$

where *y* is the cumulative number of patients who are free of symptoms at time = *t*, *y*_0_ is the cumulative number of patients who are symptom-free at *t* = *∞*, *a* is a preexponential constant, and *k*_decay_ is the rate constant for the decay of this response. SigmaPlot (v. 11.0, 2008, Systat Software, Inc. Chicago, Ill, USA) was used to conduct a least-squared fit for the values of *y*_0_, *a*, and *k*_decay_. The 95% confidence intervals for *y* values were also calculated. This equation is commonly used for simulating the decay rate of a reactant from a single model compartment.

### 2.3. Nomenclature

The FDA has approved generic, nonproprietary names for commercial formulations of botulinum toxin [22–24]. For botulinum toxin type A, BOTOX (Allergan, Calif, USA) is onabotulinumtoxinA, DYSPORT (Ipsen Biopharm Limited Co., UK) is abobotulinumtoxinA, and Xeomin (Merz Pharma GmbH & Co KGaA., Germany) is incobotulinumtoxinA [25]. For botulinum toxin type B, MYOBLOC/NeuroBloc (Solstice Neuroscience, Inc., Pa, USA; Eisai Ltd., UK) is now rimabotulinum toxin B [26]. The changes in nomenclature emphasize the different potencies and the noninterchangeable unit dosages of these distinct brand name products. As reviewed by Alberto [24], these distinctions in names emphasize the differences in manufacturing and formulation techniques that may contribute to differences in the pharmacokinetics, efficacy, safety, and antigenicity among these products.

In the present paper, partially purified toxin with nontoxic or accessory proteins is referred to as botulinum toxin type A to distinguish it from botulinum neurotoxin A (BoNT/A), the pure holotoxin.

### 2.4. Sources of Error and Uncertainty

The lists of selective keywords are not intended to be exhaustive but serve as a starting point for the present work. Additional terms, such as proctology and coloproctology [27], can be used in more comprehensive studies.

Software development involves verification and validation. Verification confirms that, for example, the equations being coded are producing the correct calculations. Software can be validated when it can model the results that best fit existing data, and is subsequently reinforced by further data obtained experimentally. For kinetically related clinical problems, the underlying processes that need to be included are still uncertain. Furthermore, mathematical models are not designed to replace validation by basic research experiments or clinical observations. Rather, models are meant to enhance validation procedures by providing stimuli for new ideas, hypotheses, and perspectives on the problems being examined.

## 3. Results and Discussion

### 3.1. Literature Search for Botulinum Toxin A and Wound Healing

A preliminary search of botXminer, using the query “wound AND (heal OR healing)” for years 1980–2010, returned over 150 citations about half of which were true positives due to the large number of references related to tetanus (false positives). From the list of true positive citations, additional keywords were identified.

A variety of indications were found, in which botulinum toxin A has been used for wound healing and related conditions. These examples included experimental cutaneous scars in animal models [15, 28] and in clinical studies: chronic anal fissures [29, 30], cleft lip surgical repair [31], traumatic head lacerations or elective excisions of forehead masses [32], focal fold granuloma [33], hypertrophic scarring [34], pressure ulcers [35, 36], Raynaud's phenomenon (vasospastic ischemia of the digits, digital ischemia, including chronic ulcers) [37], and self-mutilation injuries in Lesch-Nyhan syndrome [38]. Another healing application of botulinum toxin A, referred to as protective ptosis, is used against persistent corneal ulcers, burns, and other ophthalmic-related problems [39, 40].

**Figure 1:**
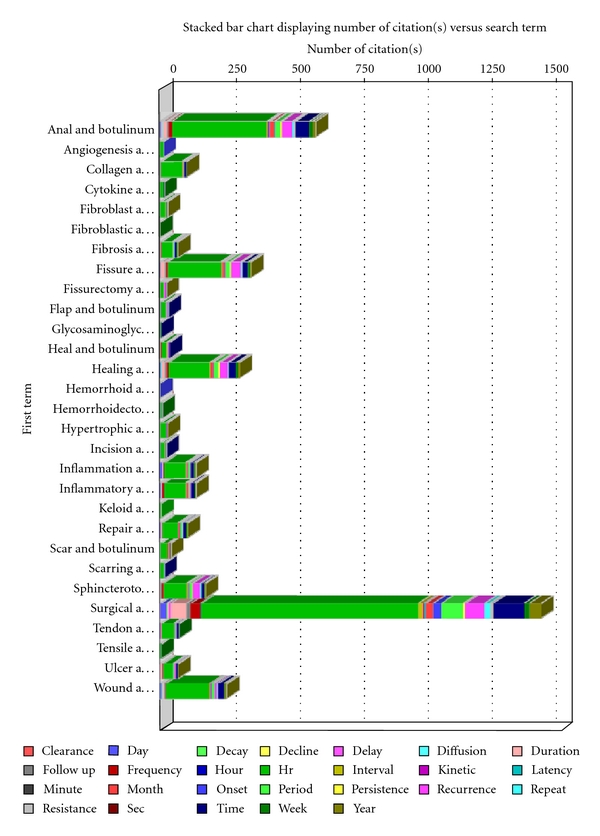
Example output from a botXminer database search. The numbers of botulinum toxin citations are shown for each of the combined 29 wound-healing (first term) and 26 time-related terms (legend) that were associated with 671 unique citations. The wound-related keywords associated with “botulinum” are associated with histograms that represent the different numbers of articles found in these multiple searches. The color-coded, time-related keywords used in these searches are simultaneously displayed in these histograms. Each “a…” in the left-hand labels signifies “and botulinum.”

Conducting a matrix batch search in which “tetanus” was filtered out, using 26 time-related terms along with the 29 wound healing-related terms, yielded 671 unique citations. From this filtered output, we concentrated on references dealing with wound conditions and indications in which botulinum has been used for therapeutic purposes in which some mechanistic, dosage, and/or kinetic information was also available (Methods, Figure 1).

### 3.2. Botulinum Toxin A and the Components of the Healing Process

The normal wound-healing process has been described as being comprised of four overlapping phases: haemostasis, inflammation, tissue proliferation, and remodeling [41]. If any of these processes are disrupted, healing is impeded leading to a chronic wound state. The interference by botulinum toxin in reducing muscle movement has helped to define healing phases further. For example, a vicious cycle involving inflammation, pain, and muscle spasm was first noted to be the underlying cause for the development of chronic anal fissures [29, 42]. Subsequently, low blood flow and ischemia were added to this cycle [43–45]. Additional components of the healing process have been identified for other conditions as presented in this section.

Scar formation is a hallmark of wound healing, and it usually causes significant physical, psychological, and cosmetic problems. Hypertrophic scarring is a common, refractory dermal disease that is manifested by the abnormal appearance of wound healing which can be the result of different types of injuries [34, 46, 47]. In the study by Xiao et al. [46], 19 patients were treated once a month over 3 months with 1.8–35 U botulinum toxin A (Hengli/CBTX-A, Lanzou Biochemical Co., China [48]). Improvements in wound healing were based on subjective grading by patients and plastic surgeons. Scores were assigned before treatment for the associated erythema, pliability (lesion softening), and the severity of itching. After a 6-month followup of the 19 patients participating in that study, 15 gave an overall assessment of their lesion improvement as “good,” and seven others rated their improvement as “excellent.” Some critical comments provided by the authors included the lack of control subjects, the study was not double blinded, and a small patient population size. Finally, there was only a relatively short follow-up time of 6 months so that no determination of the total time course of toxin action could be established.

A number of quantitative parameters may be accessed to evaluate the effectiveness of wound healing by botulinum toxin A. Increased metabolic activity and inflammation during the healing process induce muscle contractions around the edges of the skin wound [32, 46]. The major role of botulinum toxin A in this healing process is to prevent the repeated, small contractions that produce “microtraumas” near the hypertrophic scar and thereby decrease the tensile force (muscle activity) during scar formation. Traditional surgical techniques that align incisions along Langer's lines do not prevent repeated contractions [15]. The development of fibrosis also involves the deposition of extracellular collagen and glycosaminoglycans that can cause the scar to hypertrophy, invert, and become hyperpigmented resulting in poor color matching of this tissue with the neighboring skin [15, 28]. Other parameters for wound healing include size of wound, amount of and infiltration of inflammatory cells, blood vessel proliferation, and wound thickness [28].

Additional cellular and molecular mechanisms of healing by the formation of scar tissue (traumatic cicatrisation) are beginning to be elucidated [46]. Transforming growth factor *β*1 (TGF- *β*1) is a fibrotic cytokine that stimulates cellular growth, differentiation, and adherence and leads to the collagen deposition. This cytokine initiates these processes by extracellularly binding to a coupled pair of serine-threonine kinases. On binding, one receptor recruits and phosphorylates the other. This signaling pathway eventually stimulates transcription of the collagen gene and the formation of hypertrophic scars. Because human fibroblasts derived from hypertrophic scars overexpress and secrete TGF-*β*1, another wound-healing effect of botulinum toxin A has been speculated to be inhibiting the secretion of TGF-*β*1 [47]. Similarly, suramin, an antifibrotic polysulfonated naphthylurea compound, has been reported to promote wound healing by antagonizing TFG-*β*1 in muscle-derived fibroblasts [49].

The circulatory system is also affected by the apparent ability of botulinum toxin to enhance wound healing. Using a rat model for wounds, Yoo's group [50] observed that pretreatment with botulinum toxin A increased dorsal skin flap survival and concluded that this process was caused by increased perfusion. Because this toxin inhibits secretion of norepinephrine from sympathetic vasodilator and vasoconstrictor neurons [51, 52], the effect of botulinum toxin A may involve increased perfusion by decreasing sympathetic vasoconstriction in the skin flaps, thus promoting skin flap survival.

### 3.3. Kinetic Data for Onset and Duration of Healing Effects Produced by Botulinum Toxin A

The protective effects of botulinum toxin-induced ptosis have been used for the conditions of recalcitrant corneal ulcers and other surface disorders as an alternative to the surgical practice of partially sewing the eyelids together (tarsorrhaphy) [39]. This secondary healing effect is produced when botulinum toxin A is injected into eye muscles, typically the levator palpebrae superioris (LPS) muscles [53]. This therapeutic application has been the subject of studies that have also generated kinetic data [39]. From an open-label, multicentered study with 16 ophthalmic patients who received 5 U BOTOX in the LPS muscle, the time to “suitable” ptosis was 4.0 ± 0.5 days (mean ± SE, range: 2–8 days), and the duration of this ptosis was 46.0 ± 12 days (1–206 days) [39]. A similar number of patients who received a single, lower dose (2.5 U) had a comparable mean time to ptosis (3.6 days) and a shorter, mean duration (16 days). Diplopia was the only adverse effect experienced by five patients. Although statistical analyses with more patients are required to make more definitive conclusions, these trends are not unexpected but are, nevertheless, remarkable because a twofold change of a low dose apparently resulted in appreciable differences in duration.

### 3.4. Kinetic Analysis of Recurrence of Symptoms

Chronic anal fissure (CAF) is a painful condition caused by spasms of the internal sphincter smooth muscles. The traditional surgical approach has been sphincterotomy that can result in the adverse effect of incontinence [30]. Traditional nonsurgical approaches that have been used include sitz bath, topical anaesthesia, nitroglycerin, isosorbide dinitrate, nifedipine, Diltiazem, L-arginine gel, hyperbaric oxygen, and botulinum toxin A [43]. The first use of botulinum toxin as a medical alternative for CAF was conducted by Jost and Schimrigk [29]. For this indication, we chose what has been described as one of the longest follow-up studies using botulinum toxin (type A) in the treatment of CAF [54].

**Figure 3:**
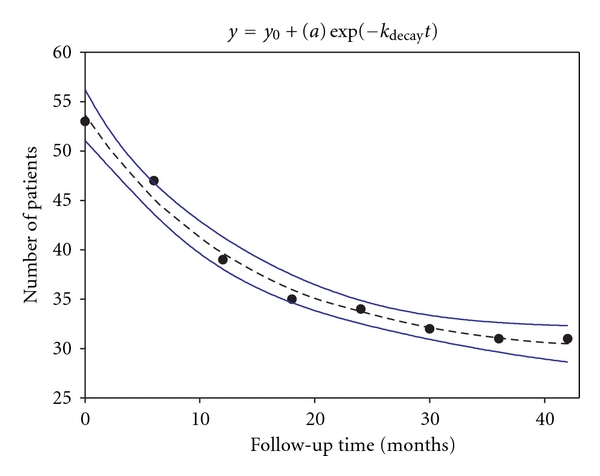
Time course of recurrence of CAF symptoms in some patients (22 of 53) in a 42-month follow-up study [55]. Initially (*t* = 0, i.e., 6 months after injection), all 53 patients were symptom-free for 6 months after injection of 10–20 U BOTOX. The fitted values from (2) (see text) are *y*_0_ = 29.2, *a* = 24.4, and *k*_decay_ = 0.85 years^−1^. The *y*-axis represents the number of symptom-free patients. Dashed line is fitted data; solid lines are 95% C.I.

We analyzed data for the time course of recurrence of CAF symptoms in patients in a 42-month follow-up study [55, Table 1]. As illustrated in Figure 3, at the beginning of the followup (at *t* = 0), all 53 patients were symptom-free for 6 months after one to two injections of 10–20 U BOTOX. Subsequently, 22 of 53 patients showed a recurrence of symptoms. Most patients (31 of 53) did not undergo recurrence indicating that they remained healed during this follow-up period. From the nonlinear fit of these data to (2), the fitted values ± SEMs are *y*_0_ = 29.2 ± 1.2, *a*  = 24.4 ± 1.3. The *k*_decay_ (decay rate constant) is 7.08 × 10^−2^ ± 1.07 × 10^−2^ month^−1^, 1.64 × 10^-6 ^min^−1^ (see below), or 0.85 years^−1^ (see Table 1). The value of 1/*k*_decay_ or *τ* is 14.1 months, and the corresponding value of *t*_1/2_( = *τ*ln⁡⁡2) is 9.8 months or 294 days.

The long-term success rate was only 31/53 (58%) or a 42% rate of recurrence [30]. From Arroyo's group [54], recurrence rate of 12% occurred initially, and a rate of 53% at the 3-year follow-up point. Bilateral fissurectomies of the internal anal sphincter combined with toxin injections [56] have been reported to have a high success rate although the follow-up time in that study was limited to 1 year. Also, it was not determined whether surgery itself produced a similar rate of healing.

**Figure 4:**
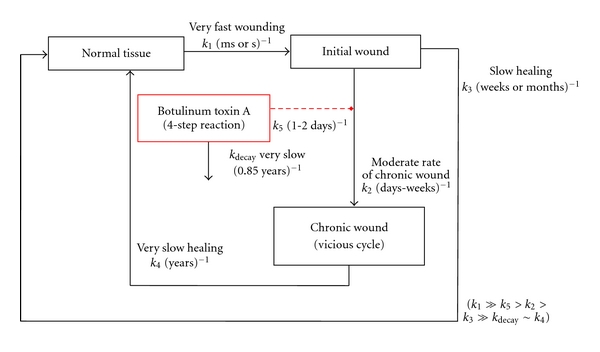
A minimal kinetic model for enhanced wound healing with botulinum toxin. Wounding of the normal tissue state is assumed to occur most rapidly leading to an initial wound state of the tissue. Healing from a significant wound is typically a slow process taking weeks or months. In the meantime, without treatment, this system enters, at a moderate rate, into a vicious cycle state (Figure 2) from which its escape represents a very slow healing process. With botulinum toxin treatment, this cycle is blocked thus allowing the tissue to return to the normal state by the slow healing route. The four-step reaction of toxin binding, translocation, internalization, and toxicity is described elsewhere [19]. A competing reaction is depicted as the slow decay or the inactivation rate of this toxin (*k*_decay_) that leads to a recurrence of symptoms.

An interpretation of the single exponential decay curve during the 42-month followup (Figure 3) is that it reflects a zero-order elimination or inactivation step of the persistent, intraneuronally located toxin [57]. This step is described by the rate constant of decay, *k*_decay_, in Figure 4 and *k*_*e*_ in a previous publication [58]. The table summarizes the processes displayed in Figure 4 and highlights the recurrence of symptoms that may be used to gauge the slow elimination or inactivation of the type A botulinum toxin. Notably, this rate may be comparable to the slow rates of healing of some chronic wounds.

Alternatively, the long-lived toxic effect may be related to the rate of restoration of intact SNAP-25 intracellular levels or a combination of a slow degradation and a persistent inhibitory action of the SNAP-25 BoNT/A cleavage product [59]. The value of *t*_1/2_ of 294 days is comparable to the recurrence times of 444 ± 132 days (mean ± S.D.; range 270–718 days [60]) for achalasia patients after receiving a single injection of 80 U BOTOX. On the other hand, our calculated decay rate constant of 1.64 × 10^-6 ^min^−1^ is about 1000 times slower than the estimated rate constant for decay of efficacy (1.1 × 10^-3 ^min^−1^) in a single dystonic patient [58, 61, 62]. This difference may be due to several factors, among which are the different patient populations, the muscles being injected, the conditions being treated, and the assessment methods.

## 4. Future Directions

While the above studies show encouraging trends in support of using botulinum toxin A in wound-healing paradigms, additional studies are necessary. Overall, more prospective clinical studies of these treatments with botulinum toxin A are needed. Evidence from blinded, randomized, placebo-controlled, multicentered studies will help determine if these toxin treatments have significant benefit and if the minimal adverse reactions can be sustained.

Future trials should also use larger populations of more homogeneous (standardized) patients and control subjects, plan to examine long-term outcomes, and conduct cost-benefit analyses [63]. Although randomized controlled trials are considered the gold standard of clinical research [64], assessing them using criteria for standardizing phase III trials remains a substantial challenge [65]. Moreover, there is also a need for additional controlled studies to clearly establish an advantage of botulinum products over other methods.

Retrospective meta-analysis studies are also needed for all of these new treatments. An outcome of one of these analyses was the low probability that type A botulinum toxin or calcium channel blockers were found to be more effective in treating CAF than nitroglycerin ointment in 182 patients [30]. Another example is Shao et al.'s analysis [44] which determined that for 279 CAF patients the traditional surgical procedure of lateral internal sphincterotomy (LIS) was more effective than BOTOX in healing chronic anal fissure. While LIS produced a higher rate of minor anal incontinence, botulinum toxin was associated with a higher rate of recurrent disease. For those patients who had a high risk of incontinence, local injection of the toxin was considered appropriate.

Computational modeling and simulation studies at different levels of granularity (i.e., detail) should also be beneficial. Starting with existing minimal kinetic models [19, 66], dose-dependent kinetic models could be developed to predict the time course (onset, duration, and recurrence of symptoms) and the extent of botulinum toxin A's effectiveness. Kinetic models could help to identify what research gaps exist and which ones can be experimentally or clinically resolved. One gap that could be experimentally verified is to determine if the intracellular diffusion of botulinum toxin A [67] is influenced by other coinjected materials, for example, epinephrine and local anesthetics (lidocaine, Xylocaine), compounds that have been considered in controlling local diffusion and predicting the extent of this toxin's paralytic effect [15, 32].

As more realistic physiological-pharmacological models are developed, more free parameters and more sources of error, assumptions, and caveats will need to be evaluated. Potentially confounding factors associated with wounds include cell viability, alkali conditions [41], the generation of reactive oxygen species, and inflammation that is related to low blood flow and ischemia [45]. Changes in the ion flow through transmembrane channels and in metabolism could also have roles in wound healing. Another confounding factor is the well-known, persistent muscle weakness (up to 5 years) that results from protracted patient immobilization due to critical illness polyneuropathy or myopathy [68, 69]. This persisting weakness and residual neurologic deficits are likely due to denervation, combined with catabolic muscle wasting and potential myopathic changes [68]. It remains for future studies to differentiate these physiological deficits from botulinum toxin-induced effects in either a botulism patient or with therapeutic treatments with this toxin.

The tissues involved in the healing process in cytoskeletal architectures and in membrane structures associated with various organs also need to be considered. Neurophysiological abnormalities, such as transient denervation-induced muscle fibrillations and presynaptic alterations producing muscle fasciculations, may also need to be considered. Other complicating factors in the healing process could also involve the biomechanical dynamic properties of soft tissue (e.g., stiffness) in response to stress and strain, and tissue anisotropy (directionality of nonhomogeneous material).

## 5. Summary/Conclusions

This succinct review examines the soft tissue wound-healing properties of botulinum toxin. When viewed from the perspective of treating neurologic and other disorders, it is noteworthy that the efficacy of this toxin is predicted to be transient as the toxin's effect wanes, while for wound healing, a permanent resolution is expected. Further, with respect to wound healing, the concept is described that for some lesions, botulinum toxin interferes with the vicious cycle of muscle spasm, pain, inflammation, decreased blood flow, and ischemia [43]. A reaction pathway scheme is outlined to illustrate botulinum toxin's involvement in stopping the vicious cycle. A minimal kinetic scheme for healing chronic wounds is also presented that includes different macroscopic states of soft tissue conditions (normal, initial wound, and chronic wound), and quantitative estimates for the relevant rate constants are provided. A definitive validation of the results, that is, the minimal kinetic model for predicting the beneficial effects of type A toxin, awaits additional clinical data. Perhaps the most useful outcome is that our kinetic model is capable of identifying a measurable gap (decay rate of toxin's effect) in our attempt to comprehend how complex, interacting biological systems respond to environmental stressors.

## Figures and Tables

**Figure 2:**
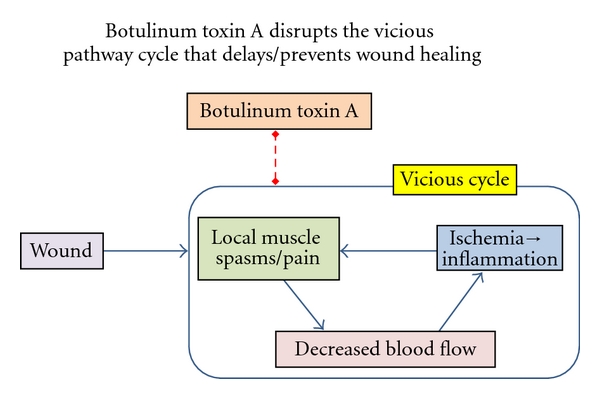
Botulinum toxin is depicted to disrupt the vicious pathway cycle that delays or prevents the healing of moderate wounds. This cycle involving spasm, pain, diminished blood flow, ischemia, and inflammation is blocked by the inhibition of chemical transmission at neuromuscular junctions by botulinum toxin A [29, 42, 43]. Dashed line is inhibitory action.

**Table 1:** Summary of processes involved in wound healing as described in text. Recurrence of symptoms results from long-term observations [55] was analyzed in the present paper and is highlighted in bold.

Parameter	Relative rates	Rate constant symbol	Units	Value
Onset of initial wounding	Very fast	*k*_1_	msec^−1^ or sec^−1^	N.A.^a^
Development of chronic wound	Moderate	*k*_2_	days^−1^ or weeks^−1^	N.A.
Healing of acute wound	Slow	*k*_3_	weeks^−1^ or months^−1^	N.A.
Healing of chronic wound	Very slow	*k*_4_	years^−1^	N.A.
**Recurrence of symptoms [55**]**, [Figure 3] **	**Very slow**	**k**_**decay**_	**years^−1^**	**0.85**
4-step reaction [19]				
Diffusion toxin to receptors	Moderate	*k*_5_	min^−1^	0.001
Binding of toxin	Fast	*k*_*B*_	min^−1^	0.058
Translocation of toxin	Fast	*k*_*T*_	min^−1^	0.141
Toxic reaction (lysis)	Fast	*k*_*L*_	min^−1^	0.013

^a^ N.A., because data are not presently available, order of magnitude estimates are given.
